# Biogas Production Potential of Thermophilic Anaerobic Biodegradation of Organic Waste by a Microbial Consortium Identified with Metagenomics

**DOI:** 10.3390/life12050702

**Published:** 2022-05-08

**Authors:** Lyudmila Kabaivanova, Penka Petrova, Venelin Hubenov, Ivan Simeonov

**Affiliations:** The “Stephan Angeloff” Institute of Microbiology, Bulgarian Academy of Sciences, Acad G. Bonchev Str., Bl. 26, 1113 Sofia, Bulgaria; pepipetrova@yahoo.com (P.P.); vhubenov7@gmail.com (V.H.); issim@abv.bg (I.S.)

**Keywords:** biogas, renewable energy sources, anaerobic biodegradation, thermophilic conditions, metagenomics

## Abstract

Anaerobic digestion (AD) is a widespread biological process treating organic waste for green energy production. In this study, wheat straw and corn stalks without any harsh preliminary treatment were collected as a renewable source to be employed in a laboratory-scale digester to produce biogas/biomethane. Processes parameters of temperature, pH, total solids, volatile solid, concentration of volatile fatty acids (VFA), and cellulose concentration, were followed. The volume of biogas produced was measured. The impact of organic loading was stated, showing that the process at 55 °C tolerated a higher substrate load, up to 45 g/L. Further substrate increase did not lead to biogas accumulation increase, probably due to inhibition or mass transfer limitations. After a 12-day anaerobic digestion process, cumulative volumes of biogas yields were 4.78 L for 1 L of the bioreactor working volume with substrate loading 30 g/L of wheat straw, 7.39 L for 40 g/L and 8.22 L for 45 g/L. The degree of biodegradation was calculated to be 68.9%, 74% and 72%, respectively. A fast, effective process for biogas production was developed from native wheat straw, with the highest quantity of daily biogas production occurring between day 2 and day 5. Biomethane concentration in the biogas was 60%. An analysis of bacterial diversity by metagenomics revealed that more than one third of bacteria belonged to class *Clostridia* (32.9%), followed by *Bacteroidia* (21.5%), *Betaproteobacteria* (11.2%), *Gammaproteobacteria* (6.1%), and *Alphaproteobacteria* (5%). The most prominent genera among them were *Proteiniphilum*, *Proteiniborus*, and *Pseudomonas*. Archaeal share was 1.37% of the microflora in the thermophilic bioreactor, as the genera *Methanocorpusculum*, *Methanobacterium*, *Methanomassiliicoccus*, *Methanoculleus*, and *Methanosarcina* were the most abundant. A knowledge of the microbiome residing in the anaerobic digester can be further used for the development of more effective processes in conjunction with theidentified consortium.

## 1. Introduction

Today’s civilization faces growing energy and environmental problems related to the depletion of fossil energy sources [1]. Yet society’s energy needs continue to increase due to fast economic growth and, therefore, much attention needs to be paid to the utilization of renewable resources for the production of valuable products and energy [2]. Renewable energy sources are part of the earth’s physical structure and are constantly being renewed by natural means so they cannot deplete [3]. The use of biofuels is highly beneficial from an ecological, economic and strategic point of view, as it significantly reduces CO_2_ emissions to the atmosphere [4]. Biomass energy is generated or produced by living or once-lived organisms. Biomass is assumed to be a renewable material because plants continue their growth after being harvested, thus the available stock remains without decreasing in addition to the incorporated carbon quantity over time. Biomass energy can be produced from plants, trees, crop residues, and other agricultural waste [5].

Globally, increased biogas production will favor energy supply through the use of renewable alternatives [6]. Lignocellulosic biomass is one of the most abundant renewable resources not only in the world, but also Bulgaria. One method of utilization is its involvement in anaerobic degradation accomplished by microorganisms. Anaerobic digestion technology uses microorganisms to utilize waste and produce methane, which could serve as a source of clean renewable energy. Temperature and substrate composition are among the main factors affecting the performance and stability of anaerobic digestion processes [7,8]. Anaerobic digestion of lignocellulosic biomass provides an excellent opportunity to convert abundantly available bioresources into energy [9]. Straw is an agricultural residue as a result of the production of rapeseed or sunflowers. It is a promising lignocellulosic substrate with a beneficial greenhouse gas balance during biofuel production [10]. Wheat straw is a complex material. Mechanical fractionation and grinding can represent a promising and efficient pretreatment of raw lignocellulosic materials for bioconversion without the addition of water and unnecessary effluent production [11]. Wheat straw must undergo several steps for conversion into biogas. The consecutive pathways include hydrolysis to produce simpler and soluble materials, acetogenesis to produce VFA, and methanogenesis to produce biogas/biomethane [12]. According to the national long-term program to encourage the use of biomass (Sofia, Bulgaria), the total quantity of wheat straw in Bulgaria is 2,714,500 t/year, of which 542,900 t/year remains unused and available [13]. Anaerobic digestion for biogas/biomethane production is an approach with environmental benefit, combining waste disposal with energy production [14]. This technology favors nutrient recycling and increases the stability of the energy supply [15]. Conducting the process of anaerobic digestion under thermophilic conditions is known to strongly affect the performance of biogas digesters leading to increased hydrolysis rates. Elevated temperature accelerates biochemical processes, enabling faster degradation and higher biogas yields from a wide variety of substrates, compared with mesophilic anaerobic digestion [16]. There is an insufficient availability of data in the literature comparing mesophilic and thermophilic treatments. There is a report on spent animal bedding consisting of feces and straw [17]. Böske et al. [18] used a continuous up-flow anaerobic solid-state reactor to treat spent horse bedding, whereas Gómez et al. [19] used a dry batch system to digest spent cow bedding. Under thermophilic conditions, the first authors observed a higher methane yield than at mesophilic temperature, while the second authors reported a lower methane yield at increased temperature. Therefore, the operating parameters of anaerobic digesters and the methods of acceleration and optimization used to improve process efficiency remain of utmost importance. Further intensive research must be carried out in the field of renewable energy generation to solve such a global dilemma.

The aim of this study is to evaluate the biogas/biomethane production potential of a biotechnological process in a laboratory-scale bioreactor, together with identification of the participating microbes, influenced by the high temperature conditions.

## 2. Materials and Methods

### 2.1. Bioreactor Design and Anaerobic Biodegradation Performance

**Figure 1:**
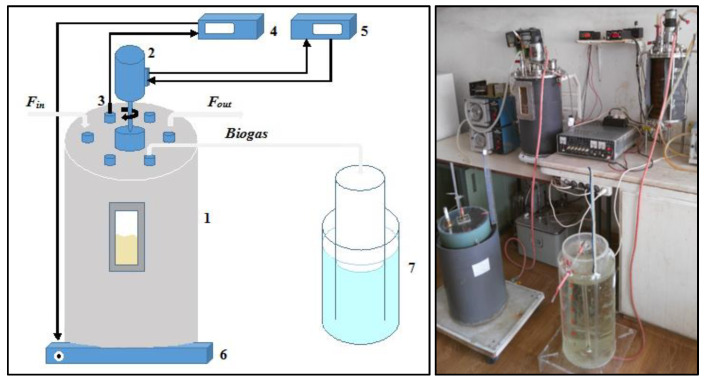
Bioreactor set-up diagram (**left**) and original experimental setup (**right**): 1. bioreactor; 2. DC-motor for stirrer; 3. Pt-100 temperature probe; 4. regulator and process indicator for temperature control; 5. regulator and process indicator for stirring speed; 6. heating device; 7. gas holder (water displacement principle).

The design consisted of a metal bioreactor (BR) with a total volume of 20 dm^3^ with a glass hatch, enabling monitoring of the level of culture fluid. Corresponding nozzles and silicone hoses were used as an inlet and outlet. During the experiments, the working volume was 14 dm^3^. The BR was equipped with a regulator for automatic temperature regulation in mesophilic (37 ± 0.5 °C) and thermophilic (55 ± 0.5 °C) modes, and a stirring system provided by a DC electric motor at a constant stirring of about 100 rpm. The inoculum used for the mesophilic process was taken from a working pilot-scale methane generating bioreactor, situated in our lab. Bacterial communities for the thermophilic process were taken from a thermophilic bioreactor, containing wheat straw, working with liquid phase recirculation. After feeding, a purge with nitrogen gas was performed to ensure an anaerobic environment. Anaerobic cultivation techniques and equipment were used. The laboratory-scale bioreactor is schematically presented in Figure 1.

Wheat straw and corn stalks were tested as substrates for the anaerobic digestion process. Prior to feeding, the substrates were mechanically pre-treated using a knife mill to decrease the particle size. The final particle size of the substrate was ≤2 mm. The air-dried portions were fed into the bioreactor manually in the following manner: about 9 L of bioreactor content was withdrawn and the solid fraction was separated from the liquid fraction using a ≈1 mm pore size sieve. The new portion of wheat straw or corn stalks was added to the liquid fraction from the previous step, and the total solids content was recalculated to correspond to 0.5% to 4.5% total solids (*w*/*v*). We assumed, using this process, that not only could the digestate water content be recycled, but also a bigger portion of microorganisms saved, together with preserving the buffer capacity of digestate. After the addition of this mixture to the bioreactor, we added the appropriate water quantity to reach the total working volume (14 L), usually about 2.0 liters of distilled water.

### 2.2. Analytical Methods

The volume of biogas produced (daily and cumulative) was measured with a graduated gas holder by method of water displacement. The composition of the biogas was determined by a specialized gas analyzer (Dräger GmbH, Lubeck, Germany) equipped with infrared sensors for the determination of methane and carbon dioxide, and term conductivity sensors for hydrogen sulfide and oxygen.

The concentration of VFA was analyzed using a Thermo Scientific Focus GC (Thermo Scientific Inc., Waltham, MA, USA) gas chromatograph, equipped with a TG-WAXMS A column (L: 30 m, I.D.: 0.25 mm, film: 0.25 mm), split/splitless injector and FID. A 1mL sample aliquot was acidified using 37% H_3_PO_4_ until pH reached 2.0, followed by centrifugation at 13,000 rpm/10 min using a Hermle Z 326K centrifuge (HERMLE Labortechnik GmbH, Gosheim, Germany). From every sample, an aliquot of 100 µL was withdrawn and mixed with an equivalent amount of internal standard solution (3,3-dimethylbutyric acid), mixed well on vortex, and 1µL was then manually injected with a microliter chromatograph syringe.

An assay for residual cellulose concentration was performed according to the photometric method proposed by Updegraff [20]. The appropriate amount of sample from the bioreactor was centrifuged at 3000 g/10 min. Briefly, the solid fraction was processed in the following manner: it was washed with distilled water and then treated with an acetic-nitric reagent for 30 min in a boiling water bath in screw-type glass tubes. After cooling to room temperature, the samples were centrifuged and the solid fraction was preserved and washed with distilled water. The resulting white precipitate (containing mainly cellulose) was dissolved in 67% H_2_SO_4_. The resulting solution was diluted with distilled water and aliquots were taken, then cooled in an ice bath, mixed with anthrone reagent, and heated in a boiling water bath for 16 min. After cooling down to room temperature, the absorbance of samples was measured at 620 nm using a Jenway 6305 spectrophotometer (Bibby Scientific, Stone, Staffordshire, UK).

Total solids were measured by dehydration of a certain volume of culture liquid at 105 °C, while volatile solids were measured by burning at 575 °C [21].

Biodegradation degree (BD) was calculated according to the following formula:

BD = [(ODMi − ODMo)/ODMi] × 100

where BD is the biodegradation degree, ODMi is the input organic dry matter, and ODMo is the output organic dry matter.

Elemental analysis with an automatic analyzer EuroEA 3000 was carried out for elements determination and C/N ratio.

Light microscopy observations were performed after Gram staining, and visualized on a Levenhuk D870T 8M (Levenhuk LTD, Tampa, FL, USA).

### 2.3. Metagenome’s Sequencing and Bioinformatics Analysis

Metagenome library construction and sequencing were performed by Macrogen Inc. (Seoul, South Korea). For library construction, total DNA was extracted from a sample using GeneMATRIX Bacterial & Yeast Genomic DNA Purification Kit (EUR_X_, Gdańsk, Poland). The preparation of the 16S metagenomic sequencing library for bacteria was performed using primer pair, which targeted the V3–V4 region (Macrogen primer set), while the archaeal metagenomic library was constructed using the primers 519F_Arch 5′CAGCMGCCGCGGTAA3′ and 806R_Arch 5′GGACTACVSGGGTATCTAAT3′ [22]. Both libraries were analyzed with a Herculase II Fusion DNA Polymerase Nextera XT Index Kit V2. The sequencing (Illumina platform) was conducted with a reading length of 301 bp and FastQC quality control. The assembly results showed that the quality-filtered data contained around 1,626,035,712 total bases, and 5,402,112 read counts for each sample. The percentage of Q20 quality reads was 94.52%.

## 3. Results and Discussion

Biotechnological processes for anaerobic digestion at 35 °C and 55 °C were performed consecutively in a laboratory-scale bioreactor. Initial experiments followed the biomethane yields for two substrates, namely, corn stalks and wheat straw as waste substrates with no chemical or biological substrate pretreatment applied, unlike as suggested by previous researchers [23,24]. Different types of pretreatment resulted, in most cases, in high investment costs. Substrates were only milled in a chopper/knife mill. Previously, for the delignification of lignocellulosic substrates, various physical, chemical, and biological pretreatment techniques were performed [25,26]. This was required due to the lignocellulosic substrates’ complexity, as the typical composition of lignocellulosic materials comprises 10–25% lignin, 40–50% cellulose, and 5–30% hemicellulose [27]. The novelty of our study was that we used untreated wheat straw, relying only on the increased temperature during the process to favor and enhance substrate swelling, decreasing the integrity, increasing the accessibility, and hence the biodegradability.

**Figure 2:**
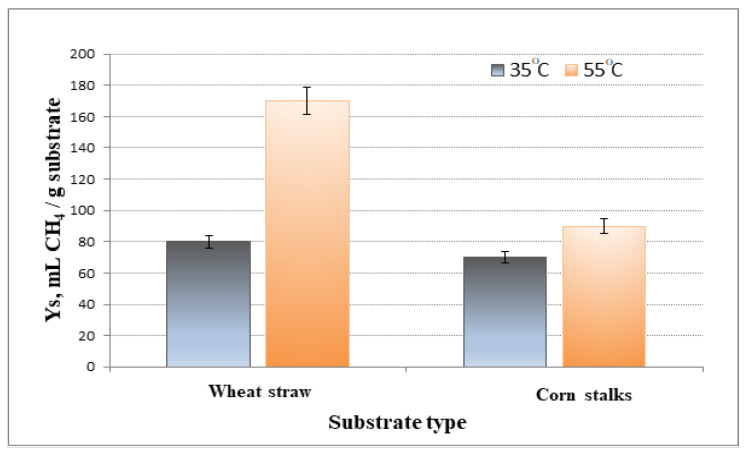
Comparison of the yield coefficients (Y_S_) for two substrate types at 35 ℃ and 55 ℃ at a constant loading rate of 5 g/L.

Two temperature regimes (35 °C and 55 °C) and two substrates were initially applied to estimate and compare the effect. For both substrates subjected to biodegradation at equal loading of 5 g/L, the volumes of biogas released were greater at 55 °C (Figure 2).

The yield coefficient was calculated. Ys is the amount of product obtained/the amount of introduced substrate. As presented in Figure 2, the increase in temperature during the anaerobic digestion process led to a significant increase in biomethane production when applying wheat straw as a substrate (52.8%) and a slight increase when using corn stalks as a substrate (21.9%). This may have been due to the higher lignin content in corn stalks (Table 1).

With an aim to break down the three-dimensional morphologic structures, modify the degree of polymerization, and increase the surface area and pore size in these complex substrates, the effect of elevated temperature is to exert its influence on one or more structural features and thus on biomass digestibility, which varies with the changes in the surrounding conditions [28]. In detail, Sorensen et al. suggested thermal acceleration of the reaction steps in four purified cellulases over a small range of temperature (10–50 °C) and substrate loads (0–100 g/L) [29]. At mesophilic conditions, the AD of lignocellulosic wastes occurs at low rates and is almost impossible without thermal, chemical or biological pretreatments [30], while at elevated temperature, these processes take place faster and with a higher biodegradation degree [31]. A possible explanation may be the better swelling of the lignocellulosic material at the higher temperature, and consequently the greater availability of the substrates for the microbial cellulase enzyme systems. Other researchers have also pointed out that thermophilic reactor performance is better, compared with mesophilic reactor performance [32].

Initial experiments involved two substrates; however, the better performance of wheat straw under the tested conditions led us to continue with that substrate, also bearing in mind that wheat straw represents the most abundant biomass available in the European Union for use in bioenergy production.

**Figure 3:**
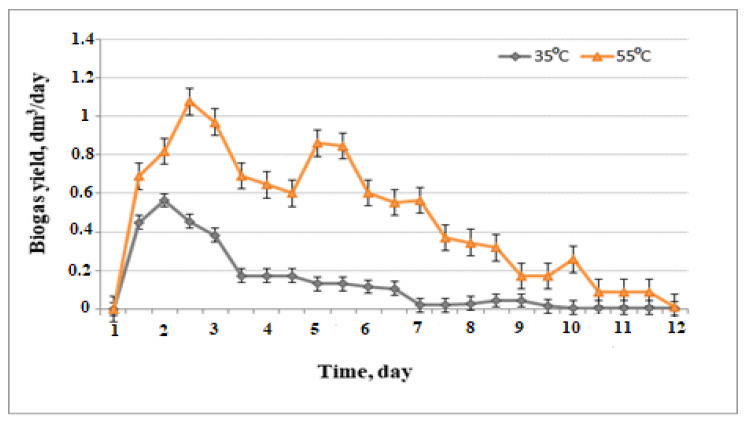
Comparison of biogas yield obtained under mesophilic and thermophilic conditions.

Biogas volume, and composition produced, were recorded daily to evaluate the process performance. The daily biogas yield using native wheat straw 5 g/L was evaluated (Figure 3).

A sharp difference was observed in biogas production at the two temperature regimes in favor of the thermophilic conditions, so the experiments were continued at 55 ℃ using wheat straw as a substrate. Moreover, a high initial biogas production rate in the thermophilic reactor was found. Thermophilic digestion was evaluated in the same way by Suhartini et al. [33] as giving higher biogas and methane productivity than mesophilic, and was able to operate in a stable manner, whereas mesophilic digestion showed signs of instability.

**Figure 4:**
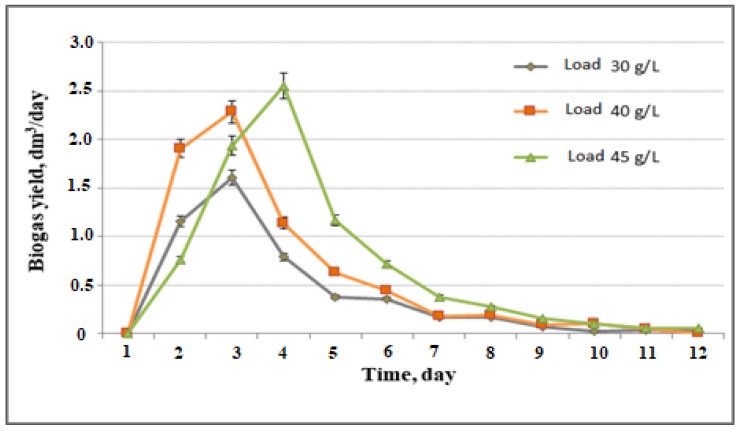
Biogas yield according to substrate loading.

The next step included estimation of the impact of loading on biogas production (Figure 4).

As seen in Figure 4, the biogas yield became higher with the increase in substrate loading. After completion of a 12-day anaerobic digestion process, the cumulative volume of biogas yield was 4.78 L for 1 L of the bioreactor working volume with a substrate loading of 30 g/L of wheat straw, 7.39 L for 40 g/L, and 8.22 L for 45 g/L. The degree of biodegradation was calculated to be 68.9%, 74%, and 72%, respectively.

**Figure 5:**
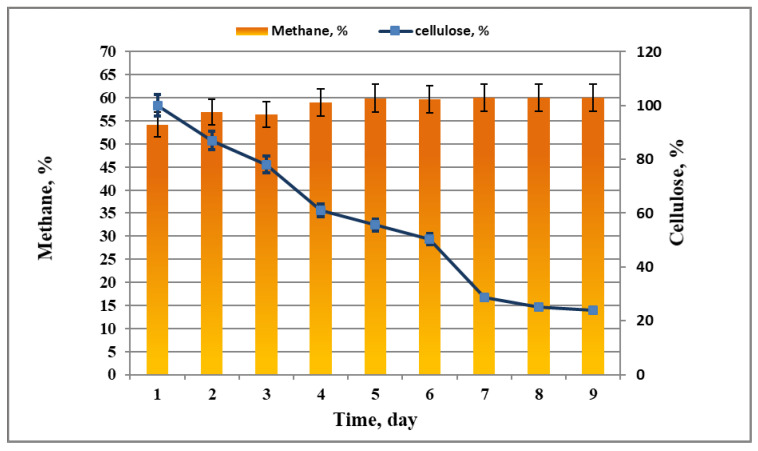
Percentage of methane measured in the biogas as a result of cellulose biodegradation.

Simultaneous with the biogas/biomethane yield increase, the quantity of residual cellulose decreased (Figure 5). The highest percent of methane measured was 60% (Figure 5). Similar results were obtained by Poh and Chong [34], who depicted an anaerobic thermophilic system of biodegradation, obtaining 64% of methane production using palm effluent.

**Figure 6:**
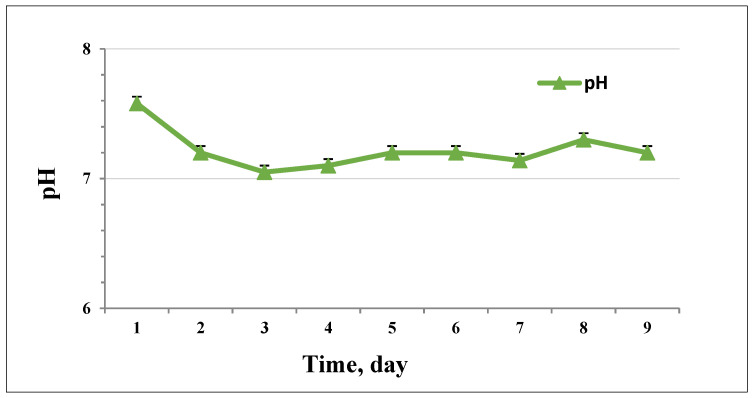
pH values during the process with 30 g/L loading.

**Figure 7:**
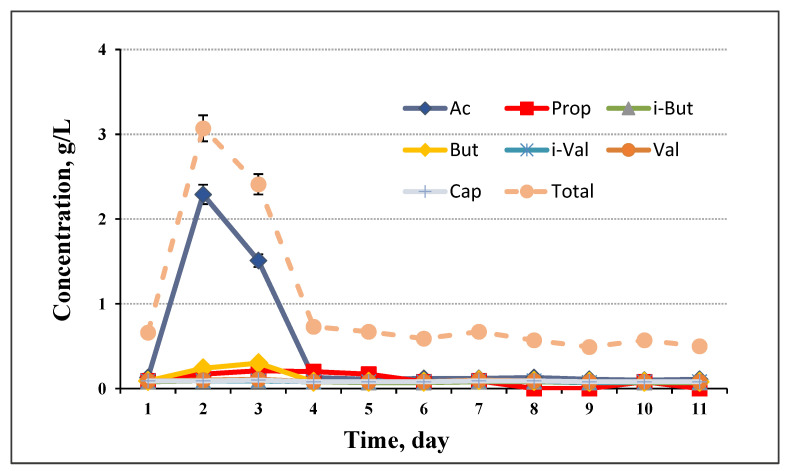
Profile of VFA during the anaerobic digestion process at 30 g/L loading.

The pH during the process of biogas production was in the range of 7.58 to 7.2 (Figure 6). With the increase in VFA concentration (Figure 7) on day 2, the pH slightly dropped. This result suggests that changes in the distribution of fermentative products could cause variation in pH and influence the process itself. The acidification in the initial phase was relatively low, possibly due to the buffering capacity of the inoculum used.

Sometimes, in digesters with low buffering capacity, changes in pH, can lead to an imbalance in the process [35]. The profile of VFA, major metabolic product in anaerobic biodegradation, was investigated. Biodegradation of cellulose leads to accumulation in the medium, mainly of acetate, followed by propionate and butyrate. In our experiments, acetate was 80% of other VFA detected (Figure 7). Speece et al. [36] stated that the average VFA concentration correlated with digester temperature, increasing from approximately 400 mg/L to more than 1000 mg/L as the temperature increased from 53 °C to 58 °C, and dropped as temperature decreased back to 53 °C. Aitken et al. [37] also reported that effluent propionate concentrations were relatively high in thermophilic digesters which operated from 51 °C to 55 °C.

The distribution profile of VFA at the other two loadings followed a similar trend. Acetate and propionate were the major VFA produced, followed by butyrate. The results in Figure 6 show that the process was not overloaded. The total VFA content was about 1.2 g/L, decreasing quickly on the second day. This demonstrated that the methane producing process was stable, and only a small amount of straw components were converted to liquid products which remained in the liquid phase.

The role of the C/N ratio is well known and reported in the literature. Most literature sources do not indicate the exact optimal value, instead an optimal range, as the C/N ratio should belong to the interval (15:1 to 30:1). Other authors have determined the ratio for anaerobic degradation of organic waste to be between 20 and 35 [38]. Practically, with all three loads, similar values were observed. Therefore, for the studied processes, the C/N ratio was in the optimal range (Table 2).

Low C/N ratio is regarded as an important factor limiting anaerobic digestion [39]. When a certain substrate has a very low C/N ratio, co-digestion with carbon-rich co-substrates is recommended, which appeared not necessary in our case. The main factors of anaerobic biodegradation of wheat straw proved that they strongly affected the performance of biogas producing bioreactors, leading to increased hydrolysis rates of thermophilic digesters. Their optimization becomes crucial for the results sought. In this study, the highest measured cumulative biogas yield was reached (8.22 L for a liter of BR working volume) at 45 g/L loading. The highest biodegradation was achieved at 40 g/L substrate loading (74%). However, conducting the processes at 55 °C led to a faster and higher degradation rate in all cases. Despite that lignocellulosic substrates are difficult to decompose, thermophilic AD showed that it can enable a higher organic loading. The accomplished process at 55 °C revealed that thermophilic systems were able to treat this type of agricultural waste at high loads. An increased methane yield was achieved for a shorter period. Accelerated metabolic rates and biogas yields from a wide variety of substrates at 50–70 °C greater than mesophilic anaerobic digestion (30–42 °C) were reported by Weiland [15].

Decreasing the viscosity, and hence the need for energy for vigorous stirring, are other important issues [40]. Therefore, we turned our attention to how much energy we would obtain from the methane obtained from the biogas mixture. It is well known that methane production using wheat straw is one of the best applications of agricultural waste [41]. In addition, biogas/methane is produced using anaerobic degradation of lignocellulosic biomass, which has a higher energy efficiency compared with ethanol production from wheat straw [42]. In addition, wheat was selected as it is the main crop of many nations; residues from agricultural activities in the European Union exceed 200 million tons every year and mostly constitute cereal straw [43].

Before discussing the energy efficiency of the studied process with only mechanical pretreatment, it is worth noting that it was carried out at laboratory-scale in a 14 L batch reactor. This methodology was useful and effective for the determination of optimal substrate pretreatment conditions and loading, and can serve as the basis for further detailed studies to be performed on full-scale devices, where the energy balance will be different in a positive way.

**Figure 8:**
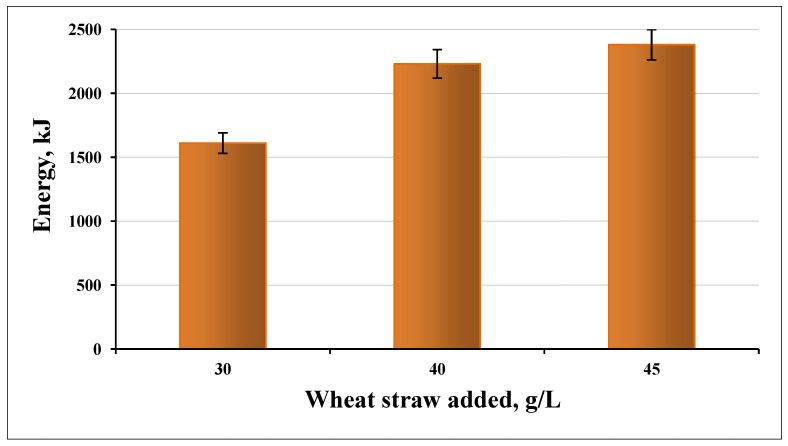
Energy yield at different substrate loadings.

**Figure 9:**
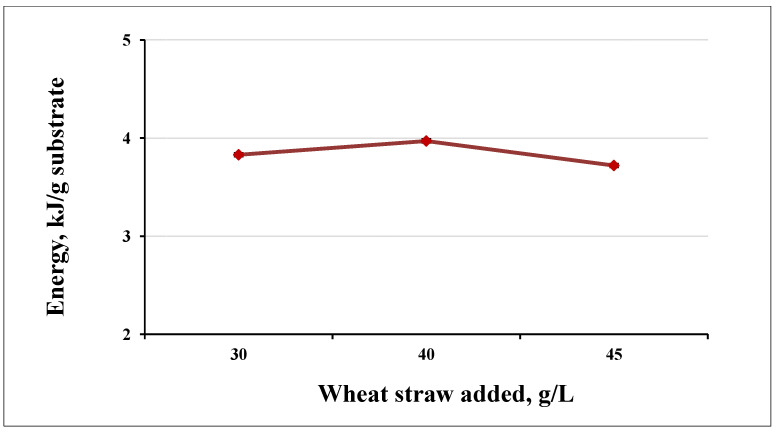
Energy yields at different loading for 1 g of substrate.

The highest energy yield was reached at a load of 40 g/L. We obtained less energy at 45 g/L per unit of substrate (Figure 8). The difference in the obtained energy between 40 g/L and 45 g/L load was smaller than between 30 g/L and 40 g/L (Figure 9).

**Figure 10:**
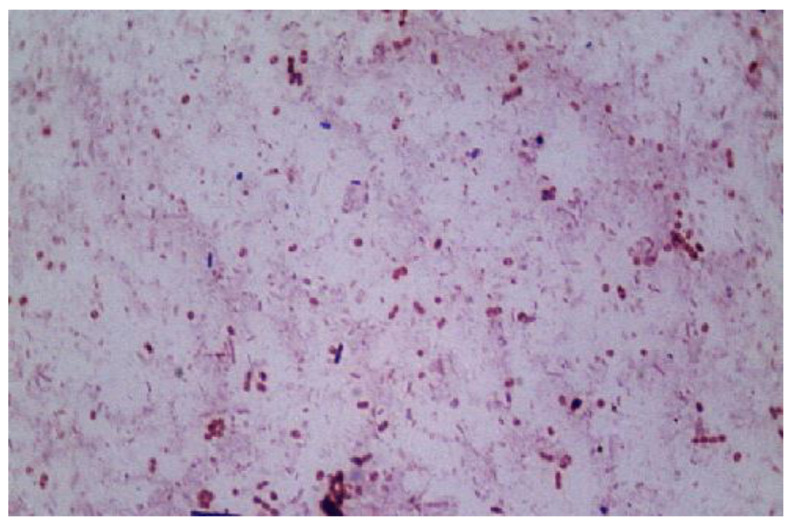
Light microscopy images obtained from the thermophilic digester (magnification ×1000).

Another important feature of the high temperature conditions was keeping away pathogens. Operation under thermophilic conditions also reduces the need for external sanitation. High temperature conditions can positively affect the pathogen elimination in the resultant digestate [44]. Figure 10 reveals a light microscopy image, obtained from our thermophilic methanogenic digester operating at 55 °C, where short rod-shaped and coccoid forms were observed. The participating microbial community had a complex structure and probably used synergetic mechanisms in the performance of anaerobic biodegradation of the strong and complex substrate, as native wheat straw was converted to energy production.

In nature, cellulose, lignocellulose, and lignin are major sources of renewable plant biomass and, therefore, their recycling is indispensable for the carbon cycle. The synergistic action of a variety of microorganisms is needed for recycling lignocellulosic materials. The AD process consists of four sequential biochemical steps: hydrolysis by hydrolytic bacteria, acidogenesis by acidogenic bacteria, acetogenesis by acetogenic bacteria, and methanogenesis by methanogenic archaea. Most essential for the last step of methanogenesis are both acetotrophic and hydrogenotrophic methanogens, but reports about their roles during this phase of the process are very limited. Microorganisms usually utilize the available carbon and nitrogen sources, which are necessarily channeled towards microbial proliferation to produce biogas and energy [45]. Temperature plays the most crucial role in the digestion rate, especially the rates of hydrolysis and methanogenesis, and determines the variety of microorganisms [46]. Metagenomics enables the comprehensive analysis of microbiomes by identifying all species participating in this complex process.

**Figure 11:**
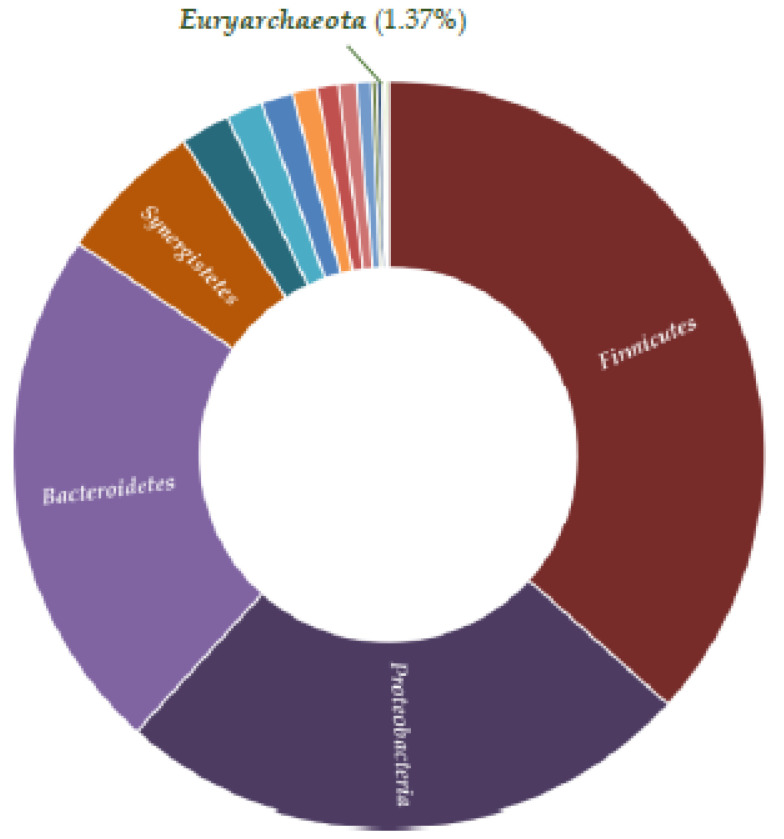
Microbial diversity in the thermophilic bioreactor (the main bacterial and archaeal phyla).

In our study, the metagenome investigation of the biodiversity in the thermophilic anaerobic digester showed that bacteria strongly prevailed over archaea, comprising 98.63% of the microbial content (Figure 11). The most abundant phyla were *Firmicutes* (38.39%), *Proteobacteria* (24.58%), *Bacteroidetes* (22.66%), *Spirochaetes* (3.92%), *Synergistetes* (2.72%), *Chloroflexi* (1.11%), *Coprothermobacterota* (0.89%), and *Actinobacteria* (0.71%).

**Figure 12:**
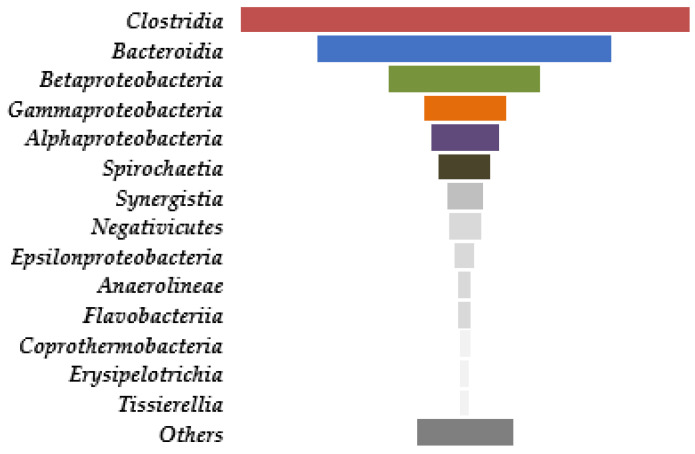
Microbial diversity in the thermophilic bioreactor.

Among bacterial classes, the most abundant were Clostridia, Bacteroidia, Betaproteobacteria, Gammaproteobacteria, and Alphaproteobacteria, followed by Spirochaetia (Figure 12).

**Figure 13:**
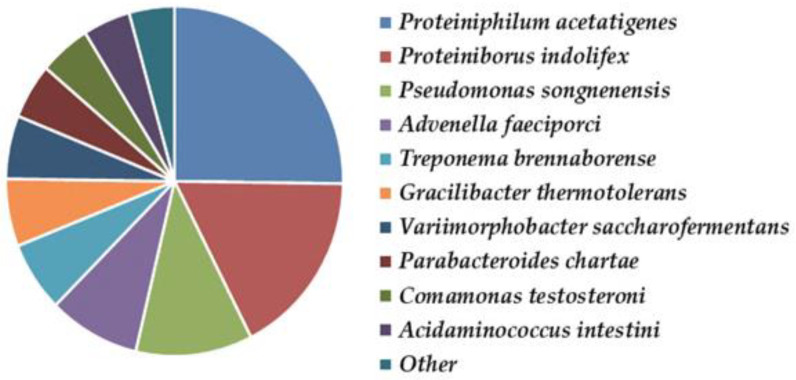
Bacterial diversity in the thermophilic bioreactor for biogas production.

Utilizing a wide range of substrates and tolerating high concentrations of VFA and alcohols, *Clostridia* were the most abundant bacteria, followed by *Bacteroidetes*, which are largely involved in biomass degradation and are indispensable in the prevention of bioreactor acidosis [47]. The prevailing bacterial genera were *Proteiniphilum* (12.25%), *Proteiniborus* (8.40%), *Pseudomonas* (5.97%), *Advenella* (4.26%), *Treponema* (3.68%), *Gracilibacter* (3.03%), *Parabacteroides* (2.94%), *Variimorphobacter* (2.84%), *Comamonas* (2.72%), *Anaerobacterium* (1.81%), *Ruminiclostridium* (1.78%), *Acetomicrobium* (1.74%), and *Thermoclostridium* (1.65%). The corresponding species are shown in Figure 13.

**Figure 14:**
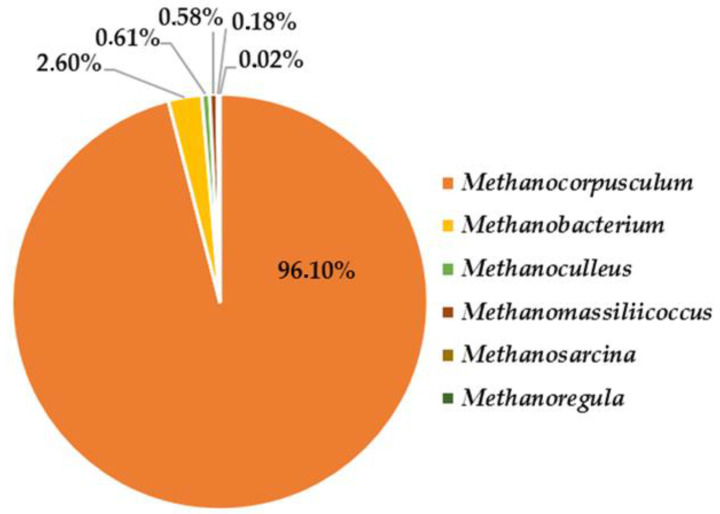
Archaeal diversity in the thermophilic bioreactor for biogas production.

Archaeal share was 1.37% of the microflora in the thermophilic bioreactor, as representatives of the genera *Methanocorpusculum*, *Methanobacterium*, *Methanoculleus*, *Methanosarcina*, *Methanomassiliicoccus*, *Methanosarcina,* and *Methanoregula* were the most abundant (Figure 14). All are members of the thermophilic phylum *Euryarchaeota*.

**Figure 15:**
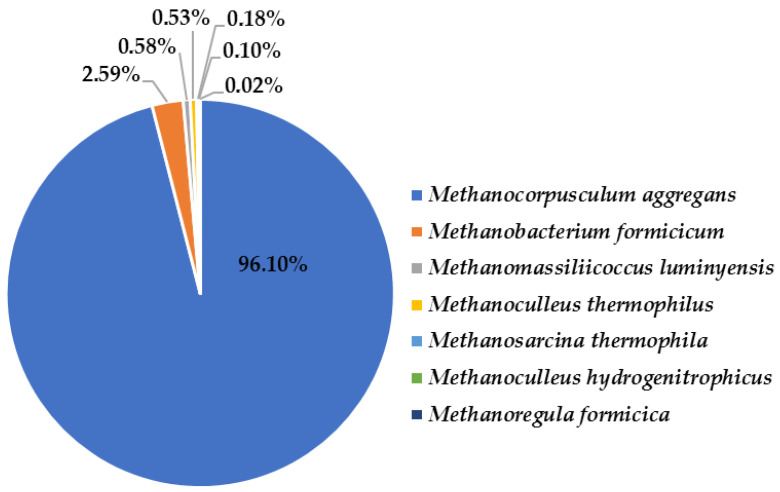
Archaeal species in the thermophilic bioreactor for biogas production.

*Methanocorpusculum* is coccoid in shape, has a temperature optimum between 30 and 40 °C, and is able to reduce CO_2_ to methane using hydrogen, formate, or alcohol. The species *Methanocorpusculum aggregans* and *Methanobacterium formicicum* (Figure 15) are rather mesophilic archaeons [48], and hydrogenotrophic methanogens, which are reported here for the first time in a thermophilic bioreactor. The presence of *Methanobacterium* (0.04%) suggested that acetotrophic methanogenesis also takes part in the bioreactor.

Reporting the presence of *Methanobacteriales* (*M. formicium*), our results are consistent with previous studies on H_2_ methane production [49,50,51]. As suggested by other authors, this order of methanogens has a selective advantage over other hydrogenotrophic methanogens in ex situ bio-methanation [52]. Thermophilic conditions can also favor coccoid hydrogenotrophic methanogens, such as *Methanosarcina* [53]. Lee et al. [54] proved that in conditions below 65 °C, microbes affiliated with methanogens dominated the population, while at higher temperatures acidogenic bacteria prevailed.

Stage-specific bacterial and archaeal populations were found to reside in thermophilic or mesophilic AD bioreactors [55]. Several metagenomic studies revealed high functional redundancy in AD bioreactors [56], explained by taxonomic variations under certain changing operational conditions, while biogas production remained relatively stable. Such a phenomenon could also explain the recovery of AD bioreactors without additional inoculation. However, the functional potential of metagenomes in AD bioreactors depends strongly on the feedstocks. Microbial communities in vegetable and fruit residues digesters have been studied [57]. This process could continue in a circle, as the resultant waste digestate after the anaerobic digestion process could serve as a biofertilizer or a cultivation medium for microalgae [58]. In this way, lowering the production cost of microalgae increases their potential for producing high-value products [59]. It was also reported that in some cases the nutrients from the digestate were more useful than other fertilizers, and digestate application and incorporation into the soil before planting could be recommended.

## 4. Conclusions

Biotechnological exploitation of lignocellulosic wastes is promising for sustainable and environmentally-friendly energy production, because of the abundant availability of these renewable sources.

Wheat straw, as agricultural waste, was involved in anaerobic digestion at elevated temperature, and biogas was obtained with biomethane as an energy carrier for further fuel production.

Conducting the process at 55 °C led to faster and higher hydrolysis rates and an increased biodegradation degree, achieved in a stable system that enabled higher organic loading.

A microbial consortium is the main tool for efficient utilization of complex waste substrates. Metagenomics was applied in the community structure elucidation, proving its complexity and probable synergetic mechanisms in the performance of anaerobic biodegradation. The most important genera were *Proteiniphilum*, *Proteiniborus*, and *Pseudomonas*, while the archaeal share of the microflora in the thermophilic bioreactor included the genera *Methanocorpusculum*, *Methanobacterium*, *Methanomassiliicoccus*, *Methanoculleus* and *Methanosarcina*. Better understanding of natural microbial communities can make their future application in energy production more felicitous.

## Figures and Tables

**Table 1:** Comparison of some components of wheat straw and corn stalks.

Parameter	Wheat Straw	Corn Stalks
TS, %	93.1 ± 0.05	95.0 ± 0.05
VS, %	88.4 ± 0.05	89.8 ± 0.05
Total nitrogen, g/L	1.1 ± 0.05	0.92 ± 0.05
Proteins, g/L	6.5 ± 0.05	4.0 ± 0.05
Cellulose, % VS	32–38 ± 0.05	26–37 ± 0.05
Hemicellulose, % VS	21–28 ± 0.05	22–29 ± 0.05
Lignin, % VS	15–20 ± 0.05	17–23 ± 0.05

**Table 2:** Elemental analysis of final solid fraction.

Samples	Element (%)		
Substrate	Carbon, C	Nitrogen, N	C/N Ratio
Loading 30 g/L	42.18	2.31	18.26
Loading 40 g/L	42.41	2.27	18.68
Loading 45 g/L	43.06	2.29	18.80

## Data Availability

Not applicable.
